# A194 IMPACT OF SUPPRESSOR OF CYTOKINE SIGNALING 1 (SOCS1) EXPRESSION IN INTESTINAL EPITHELIAL CELLS ON THE GUT MICROBIOTA

**DOI:** 10.1093/jcag/gwac036.194

**Published:** 2023-03-07

**Authors:** L M Leon, G Marrero Cofino, A Delannoy, M Placet, S Geha, E F Verdú, L -C Fortier, A Menendez, C Saucier

**Affiliations:** 1 Departments of Immunology and Cell Biology; 2 Pathology, Université de Sherbrooke, Sherbrooke; 3 McMaster University, Hamilton; 4 Microbiology and Infectious Diseases, Université de Sherbrooke, Sherbrooke, Canada

## Abstract

NOT PUBLISHED AT AUTHOR’S REQUEST

**Disclosure of Interest:**

None Declared

